# Assessment of Current Knowledge, Awareness and Attitude Towards Dental Implants as a Treatment Option for Replacement of Missing Teeth in Riyadh, Saudi Arabia

**DOI:** 10.7759/cureus.34189

**Published:** 2023-01-25

**Authors:** Abbasi Begum Meer Rownaq Ali, Taif Abdulhafith Alzaidi, Reem Yaqoub Alghimlas, May Kamal Alenezi, Yara Albesher, Haneen Abdullah Alosaimi

**Affiliations:** 1 Department of Prosthodontics, College of Applied Medical Sciences, Riyadh Elm University, Riyadh, SAU; 2 Dentistry, King Saud Bin Abdulaziz University for Health Sciences, Riyadh, SAU; 3 Department of Dentistry, Princess Nourah Bint Abdulrahman University, Riyadh, SAU

**Keywords:** treatment option, saudi arabia, replacement of missing teeth, knowledge and awareness, dental implants

## Abstract

Objective: To assess the current level of knowledge, awareness, and attitude towards dental implants as a treatment option for the replacement of missing teeth in Riyadh, Saudi Arabia.

Material and method: A random sample of 1000 Saudis (including both males and females) from Riyadh, Saudi Arabia, was selected. In accordance with research ethics codes, informed consent was obtained from research participants before approaching them via a structured online questionnaire using Google Forms; additionally, questionnaires were distributed in public places and promoted on social media to be answered anonymously. The data were coded, tabulated, and analyzed using Statistical Package for Social Sciences (SPSS; IBM Corp., Armonk, NY, USA) software. Descriptive statistics were calculated.

Results: Around more than half of the study population (56.3%) opted for dental implants as a treatment option if they had to choose among the various options, and for those who did not choose dental implants, high cost was the major factor. The Pearson correlation between dental implant information and whether it was provided by their dentists and age was significant, and the majority of those who heard about dental implants are between the ages of 30 and 50. Also, it was noted that many of the participants who were working in the government sector (49.5%) had dental implants and were aware that dental implants as a treatment option were provided by their dentist when compared to those who were working in the private sector (12.1%) and the unemployed (24.7%), and this difference was statistically significant.

Conclusion: It was also observed that there is inadequate knowledge regarding the longevity of dental implants, and participants who were working in the government sector had dental implants and were aware that dental implants as a treatment option were provided by their dentist when compared to those who were working in the private sector, and around half are not aware that dental implant treatment can be covered by insurance.

## Introduction

The field of dentistry is constantly advancing, and as new treatments to restore, remediate, or rehabilitate natural dentition are introduced, patients’ treatment options are growing by the year. One of these options is dental implants, which were proposed first in 1965 by Brenmark, who initiated experimental studies to understand and explain the concept of osseointegration [1]. Ever since that era, the fields of both implantology and dental prosthetics have garnered significant attention from the public as a fixed option to remediate the loss of dentition and become more viable. Throughout the years, an increase in dental implant prevalence has been noted in several areas in the United States of America (USA) [2]. Dental implants have become the preferred option for restoring missing teeth due to their high success rate and excellent longevity [3-5]. Additionally, dental implants in both edentulous and partially edentulous patients increased their quality of life and enhanced their masticatory functions [6]. Moreover, they have enhanced denture preservation, stability, functional effectiveness, and quality of life since they were first used to treat edentulous patients, where new age changes such as the use of zirconia implants, use of various implant modifications, digital protocols can prove to be very helpful [7-9]. Dental implants made of zirconia might be an alternative to titanium ones, which could exhibit a dark, unattractive color through the surrounding soft tissues. Zirconia has the ability to sufficiently eliminate plaque on the implant and surrounding tissues, which is essential for soft tissue healing and implant success at the bone level [9]. However, due to a lack of awareness and knowledge, some patients may overlook dental implants as a treatment option [10]. Despite the previously done studies on the knowledge and awareness of dental implants in Saudi Arabia [11], limited data is available that is specific to Riyadh with a presentable sample size. Also, previous studies have not excluded dentists or dental students, which may have given a biased result, and hence this study was conducted excluding dental specialists, dentists, and dental students.

The aim of our study was to assess the current level of knowledge, awareness, and attitude toward dental implants as a treatment option for the replacement of missing teeth in Riyadh, Saudi Arabia.

## Materials and methods

This study was submitted for ethical committee clearance to the Ethical Committee Review Board of Riyadh Elm University with Institutional Review Board (IRB) number SRP/2022/112/770/728. A random sample of 1000 Saudis (including both males and females) from Riyadh, Saudi Arabia, was selected. In compliance with the codes of research ethics, informed consent was obtained from research participants. After obtaining approval from the IRB at Riyadh Elm University, the participants were approached through a structured online questionnaire using Google Forms; also, questionnaires were distributed in public places and promoted on social media. The questionnaire was divided into three main parts.

The first section contains demographic, social, and economic data. The second part deals with more detailed questions related to the knowledge of the participants regarding dental implants. Questions related to their attitude and their willingness to learn were included in the last section.

Inclusion and exclusion criteria

All the Saudi citizens in Riyadh, aged 18 and above, were included in the study. Other nationalist dentists, dental specialists, and dental students with dental knowledge were included in the study. Validation of the questionnaire: A modified version of a pre-existing questionnaire was used. Questions are obtained from the existing research studies, and a few questions were added that were assessed for face validity by an experienced prosthodontist (Table 1).

**Table 1:** Questionnaire of the study

Demographic and Socioeconomic data		
Variable characteristic	Variable analysed	Number of respondents
Age	Below 18 years	
	18- 30 years	
	30-50 years	
	Above 50 years	
Gender	Male	
	Female	
Education level	Uneducated	
	Elementary school Middle school	
	High school Diploma	
	Bachelor Master	
	PHD	
	Others	
Nationality	Saudi	
	Non-Saudi	
Educational status	Government sector	
	Private sector	
	Self-employed	
	Unemployed	
	Others	
Profession	Dentists	
	Dental Students	
	Health Professionals (Other than dentists)	
	Teaching Professionals (Other than dentists) Business and Administration Professionals Information technology professionals	
	Financial Sector	
	Elementary Occupations-Laborer, cleaners, Mining, Construction, Manufacturing, Transport and others	
	Unemployed	
	Others	
Do you have insurance	Yes	
	No	
Knowledge and Awareness about Dental Implants		
Do you have a missing tooth	Yes	
	No	
	I don’t know	
Are you aware that various treatment options are available for replacement of missing teeth like removable appliance, fixed appliance and Dental Implants?	Yes	
	No	
	To some extent	
Have you ever heard about dental implants?	Yes	
	No	
	To some extent	
If you have a missing tooth/ teeth what treatment option will you opt for?	Removable appliance	
	Fixed appliance	
	Dental Implants	
	I will not replace it	
	Not Applicable	
If you didn’t choose dental implants as a first choice, what’s the main reason?	High cost	
	Fear of surgery	
	Fear of a foreign body in the jaw	
	Complicated treatment	
	Time consuming	
	Others	
Have you ever had a dental implant?	Yes	
	No	
According to you what is a dental implant?	Screw	
	Piece of metal	
	Heard about it, but cannot explain it	
	Never heard about it	
	Don’t know	
Where do you think implant is placed?	Jaw bone	
	Gum	
	Neighbouring teeth	
	I don’t know	
What material is Dental implant made of?	Ceramic	
	Stainless Steel	
	Titanium	
	Porcelain	
	Don’t Know	
What do you think dental implant placement means it replaces which part of teeth?	Crown	
	Root	
	Both Crown and root	
	None	
	I don’t know	
How long do you think does dental implant last?	Less than 5 years	
	5-10 years	
	10-20 years	
	More than 20 years	
	I don’t know	
Why do you think dental implants fail?	Due to patient	
	Due to dentists	
	Due to poor oral hygiene	
	Due to implant type and quality	
	I don’t know	
Do you know whether your dentist provides implants?	Yes	
	No	
	Maybe	
Who among the following are the most qualified to place dental Implants?	Oral Surgeon	
	Prosthodontist	
	Periodontist	
	General practitioner	
	All of the above	
	Don’t know	
Are you aware, if Dental implant treatment can be covered by your insurance?	Yes	
	No	
	To some extent	
	I don’t know	
Attitude towards Dental implant treatment and willingness to learn		
Do you think the replacement of missing teeth is important?	Very important	
	Somewhat important	
	Neither important nor unimportant	
	Not important at all	
Do you think dental implants need special care and hygiene as compared to natural teeth?	Much more than natural teeth	
	Same as natural teeth	
	Very little care is required	
	No special care is required	
What’s the main source of information regarding dental implant?	I don’t know	
	Dentist	
	Friends and relatives	
	Newspaper and magazines	
	Television	
	Social media	
	Internet	
	Others	
Would you like to know more about dental implants?	Definitely	
	Likely	
	Maybe	
	Definitely not	

Statistical analysis

The data were coded, tabulated, and analyzed using Statistical Package for Social Sciences (SPSS) software version 23 (IBM Corp., Armonk, NY, USA). Descriptive statistics were calculated. There is insufficient knowledge and awareness among the Saudi Arabian population about dental implants as a treatment option for replacing missing teeth in Riyadh.

## Results

Out of 1182 responses, 1135 were included after the application of the exclusion criteria. The majority of participants were female: 75.4% were female and 24.6% were male (Table 2, 3).

**Table 2:** Demographic information of the study participants

Variables		n	%
Age	<30 years	465	41.0%
	30-50 years	459	40.4%
	>50 years	211	18.6%
Gender	Male	279	24.6%
	Female	856	75.4%
Educational level	Elementary	10	0.9%
	Middle	13	1.1%
	High school	182	16.0%
	Diploma	108	9.5%
	Bachelors	715	63.0%
	Masters	73	6.4%
	Ph.D	23	2.0%
	Others	11	1.0%
Nationality	Saudi	1106	97.4%
	Other nationalities	29	2.6%
Employment status	Government sector	410	36.1%
	Private sector	166	14.6%
	Unemployed	417	36.7%
	Others	142	12.5%
Profession	Dentists	14	1.2%
	Dental Students	20	1.8%
	Health Professionals	95	8.4%
	Teaching Professionals	158	13.9%
	Business and Administration Professionals	120	10.6%
	Information technology professionals	62	5.5%
	Financial Sector	38	3.3%
	Elementary work	0	0.0%
	Unemployed	253	22.3%
	Others	375	33.0%
Do you have insurance	Yes	433	38.1%
	No	702	61.9%

**Table 3:** Profession-related characteristics of the study population

	Frequency	Percent	Valid Percent	Cumulative Percent
Dentists	14	1.2	1.2	1.2
Dental Students	20	1.8	1.8	3
Health Professionals	95	8.4	8.4	11.4
Teaching Professionals	158	13.9	13.9	25.3
Business and Administration Professionals	120	10.6	10.6	35.9
Information technology professionals	62	5.5	5.5	41.3
Financial Sector	38	3.3	3.3	44.7
Unemployed	253	22.3	22.3	67
Others	375	33	33	100
Total	1135	100	100	

Knowledge and awareness of dental implants

40.2% of the participants were aware of the availability of various treatment options for the replacement of missing teeth, like removable appliances, fixed appliances, and dental implants. More than half of the study population, around 56.3%, have opted for dental implants as a treatment option if they had to choose among the various options, and for those who did not choose dental implants, high cost was the major factor. Around 83.3% didn’t have a dental implant, which was statistically significant. Although the majority of them (51.2%) chose that a dental implant is a screw, approximately 26.2% stated that they have heard about it but cannot clearly explain what it is. Although many have responded correctly that implants are placed within the jawbone, around 26.4% don’t know where they are placed, and an alarming number of participants (63.4%) don’t know what the implant is made of. More than half of the study participants are unaware of which portion of teeth the dental implant replaces. About 57.8% of people don’t know about the longevity of dental implants, and when it was compared with the various professional results, statistically significant results were found. Also, 34.2% are not aware of the reason for the failure of dental implants, and among the various reasons implant type and quality were considered to be a major factor for implant failure, only 17.7% believe that dental implants fail due to poor oral hygiene. The difference in knowledge regarding the etiology of the failure of dental implants among the various professions is also found to be statistically significant (Table 4).

**Table 4:** Responses related to the knowledge of dental implants

Variables		n	%
Do you have a missing tooth	Yes	548	48.30%
	No	557	49.10%
	Some extent	30	2.60%
Are you aware that various treatment options are available for the replacement of missing teeth like removable appliances, fixed appliances, and Dental Implants?	Yes	456	40.20%
	No	303	26.70%
	Some extent	376	33.10%
Have you ever heard about dental implants	Yes	1012	89.20%
	No	66	5.80%
	Some extent	57	5.00%
If you have a missing tooth/ teeth what treatment option will you opt for?	Removable appliance	29	2.60%
	Fixed appliance	344	30.30%
	Dental Implants	639	56.30%
	I will not replace it	44	3.90%
	Not Applicable	79	7.00%
If you didn’t choose dental implants as a first choice, what’s the main reason	High cost	503	44.30%
	Fear of surgery	200	17.60%
	Fear of a foreign body in the jaw	55	4.80%
	Complicated treatment	61	5.40%
	Time consuming	9	0.80%
	Others	80	7.00%
	Not applicable	227	20.00%
Have you ever had a dental implant?	Yes	190	16.70%
	No	945	83.30%
According to you what is a dental implant	Screw	581	51.20%
	Piece of metal	55	4.80%
	Heard about it, but cannot explain it	297	26.20%
	Never heard about it	33	2.90%
	Don’t know	169	14.90%
Where do you think the implant is placed?	Jaw bone	628	55.30%
	Gum	186	16.40%
	Neighbouring teeth	21	1.90%
	I don’t know	300	26.40%
What material is Dental implant made of?	Ceramic	84	7.40%
	Stainless Steel	109	9.60%
	Titanium	171	15.10%
	Porcelain	51	4.50%
	Don’t Know	720	63.40%
What do you think dental implant placement means it replaces which part of teeth	Crown	70	6.20%
	Root	114	10.00%
	Both Crown and root	359	31.60%
	None	21	1.90%
	Don’t know	571	50.30%
How long do you think dental implants last?	Less than 5 years	22	1.90%
	5-10 years	104	9.20%
	10-20 years	126	11.10%
	More than 20 years	227	20.00%
	Don’t know	656	57.80%
Why do you think dental implants fail?	Due to patient	37	3.30%
	Due to dentists	131	11.50%
	Due to poor oral hygiene	201	17.70%
	Due to implant type and quality	378	33.30%
	Don’t know	388	34.20%
Do you know whether your dentist provides implants?	Yes	329	29.00%
	No	329	29.00%
	May be	477	42.00%
Who among the following are the most qualified to place dental Implants?	Oral Surgeon	418	36.80%
	Prosthodontist	196	17.30%
	Periodontist	37	3.30%
	General practitioner	7	0.60%
	All of the above	141	12.40%
	Don’t know	336	29.60%
Are you aware, if Dental implant treatment can be covered by your insurance?	Yes	117	10.30%
	No	390	34.40%
	Some extent	69	6.10%
	Dont know	559	49.30%

The Pearson correlation with respect to the information on dental implants and whether it was provided by their dentists and the age was significant, and the majority of those who heard about dental implants fall in the age range of 30 to 50 years. Also, it was noted that many of the participants who were working in the government sector (49.5%) had dental implants and were aware that dental implants as a treatment option were provided by their dentist when compared to those who were working in the private sector (12.1%) and the unemployed (24.7%), and this difference was statistically significant. The majority of the study participants are not sure if the dental implant treatment is provided by their dentist. A vast majority of the participants think that oral surgeons are the most qualified to place dental implants. Also, around half of the study population is not aware that dental implants can be covered by their insurance plan. 

Attitude toward dental implant treatment and willingness to learn

60.5% think that dental implants need special care and hygiene as compared to natural teeth. Dentists were the primary source of information for approximately 41.9% of respondents, followed by the internet (15%), friends and relatives (13%), social media (10.6%), newspapers/magazines (0.4%), and television (0.7%). The majority of them (54.6%) are willing to learn more about dental implants (Table 5).

**Table 5:** Attitude and willingness to learn towards dental implants

Variables		n	%
Do you think the replacement of missing teeth is important	Very important	776	68.40%
	Somewhat important	284	25.00%
	Neither important nor unimportant	50	4.40%
	Not important at all	25	2.20%
Do you think dental implants need special care and hygiene as compared to natural teeth?	Much more than natural teeth	687	60.50%
	Same as natural teeth	343	30.20%
	Very little care is required	67	5.90%
	No special care is required	38	3.30%
What’s the main source of information regarding dental implant?	I don’t know	168	14.80%
	Dentist	475	41.90%
	Friends and relatives	148	13.00%
	Newspaper and magazines	5	0.40%
	Television	8	0.70%
	Social media	120	10.60%
	Internet	170	15.00%
	Others	41	3.60%
Would you like to know more about dental implants?	Definitely	620	54.60%
	Likely	146	12.90%
	Maybe	251	22.10%
	Definitely not	118	10.40%

## Discussion

Since the inclusion of dental implants as a treatment option, dental practice has undergone a significant transformation. With long-term success and improved patient satisfaction, dental implants are growing in popularity as a treatment option among the general public. Riyadh, Saudi Arabia, has seen a rise in the acceptance and popularity of dental implants over the past several years. Therefore, this cross-sectional study aimed to assess and evaluate the level of knowledge and awareness of dental implants as a treatment option for the replacement of missing teeth among the Saudi population in the central region of Saudi Arabia. Our assessment included participants who exceeded the age of 18, had Saudi citizenship, and lived in Riyadh, with the exclusion of dental professionals and dental students. The inclusion of dental professionals and dental students was made with the aim of ensuring the garnering of representative responses from the general public.

Unlike previous cross-sectional studies in Saudi Arabia, our obtained data indicated that the major source of information regarding dental implants chosen by our sample is dental professionals, followed by the internet. This is opposite to the results of Abdulrahman Alajlan et al. [12], where friends and relatives were the main sources of information (38%, 31%, and 30%, respectively), highlighting a change in the public’s knowledge of the last few years. In this study, the participant’s level of awareness regarding dental implants as a treatment option was 40.2%. In previous studies done by Al-Johany et al. [13] and Al-Rafee et al. [4], which were done in Riyadh, Saudi Arabia, they found a higher level of awareness among participants (56% and 66%, respectively). In 56.5% of cases, dental implants are the preferred treatment option for replacing missing teeth. Surprisingly, 55.5% of the participants knew the proper location of the dental implant, in contrast to other studies done by Al-Rafee et al. and Al-Johany et al., in which 51% and 50.1% of the participants did not know the proper location of the dental implant, respectively [4,13].

Correspondingly with previous assessments of the public’s knowledge of dental implants, the most significant factor preventing patients from undergoing dental implant procedures is the financial cost of the procedure, followed by a fear of surgery, which is similar to Al-Rafee et al., Hosadurga et al., and Mously et al. [4,7,14]. Additionally, the participants selected dental implants as the treatment they would opt for, followed by a fixed dental prosthesis in case of partial or full dentition loss. Studies have shown that the Saudi population in Riyadh has a huge knowledge gap regarding nearly every aspect of dental implants [11,13], and the public's knowledge and awareness level regarding the usage of dental implants as a tooth replacement alternative need to be raised through educational programmes by dental care experts and specialists.

When asked about the material of dental implants, a significant number of participants (63.4%) admitted to a lack of knowledge, and 15.1% answered ceramics (7.4%), stainless steel (9.6%), and porcelain (4.5%). 29.6% of the respondents don’t know the dental professionals who are most qualified to perform such procedures; this finding is in contrast with one of the research studies done by Kinani et al. in Jazan Province of Saudi Arabia [15], where the majority of the respondents said that only specialists can place dental implants. Furthermore, the participants also failed to answer questions regarding the longevity of dental implants, highlighting a need for dental professionals to educate the general public regarding the technical, surgical, and general knowledge of dental implants [15]. It is well noted that the long-term success of dental implants depends highly on maintaining healthy tissue around the implants. The peri-implant disease can be avoided with proper dental hygiene practices, such as using soft toothbrushes, interproximal brushes, hard plastic cleaning tools, and mouth rinses. The current study population (60.5%) agreed that dental implants require more special care and hygiene as compared to natural teeth [15]. The vast majority of the general public agreed that replacing missing teeth is critical. At the same time, the majority of them (44.3% of participants) stated that the high cost of dental implants is the reason they do not choose them as a treatment option. This finding is consistent with the three studies mentioned in the dental implant literature [16-18]. The majority of them (54.6%) are willing to learn more about dental implants. In their questionnaire-based study of the Nigerian population, Ajayi et al. [19] found that dental health professionals (41.5%) were the primary sources of knowledge on dental implants, followed by friends (17.7% of the study group). Only 14.6% of the subjects demonstrated awareness about dental implants, while 35% of the survey participants lacked this expertise. Additionally, those with higher education levels knew more about implants. Other significant disadvantages of implant treatment included higher costs and more surgical requirements. The replacement of anterior teeth with implants was chosen over posterior teeth by the subjects. However, even among responders with greater education levels, a lower awareness level was seen. Additionally, implant-based prosthesis replacement was not preferred because it was more expensive and required surgical intervention [19]. In contrast to other studies where dental professionals were found to have played a significant role in disseminating knowledge about this treatment modality, Sakshi et al., in their study on knowledge among undergraduate dental students on dental implants, estimated that 99% of the study group had prior knowledge about dental implants. This knowledge was primarily gleaned from audio-visual resources and the Internet. A maximum lifespan of 10 years was reported by 55.68% of students, while a 10- to 20-year lifespan was reported by 18.75%, and a lack of knowledge about this by 26.7% of subjects, respectively. Additionally, implant pricing played a significant role in this decision to pursue implants as a kind of treatment [20]. Only 32.5% of the study subjects in Mathuriya et al.'s questionnaire-based study of dental patients in Bhopal were aware of dental implants as a treatment option, and the majority of them were unaware of the procedure's details or the benefits and drawbacks of utilising dental implants [21]. Only 25.8% of the study cohort knew anything about dental implants, according to Kumar and Chauhan's assessment of 620 Indore, India-based participants' knowledge and awareness of dental implant use. The majority of patients who chose implants as a therapy option did so because of their superior aesthetics (70%). However, because of their expensive price, 70% of people also preferred not to replace their implant-supported prosthetics [22].

In a six-month study of 220 Nigerian participants, Gbadebo et al. [23] discovered that 71.1% of the cohort had no idea what dental implants were, while just 28.9% were aware of their availability. 13.3% of study participants opposed this course of treatment, whereas 22.6% preferred implant-based prostheses. However, 61.9% of respondents were unsure about this form of treatment. Following other sources, including audiovisual media, the Internet, and peer groups as secondary sources of information about dental implants, 68% of the participants cited dental professionals as the primary source [23]. According to Pommer et al., 79% of Austrians are in favor of receiving dental implant treatment [24]. In their investigation, Chowdhary et al. only found 23.24% awareness [25]. Various researchers from throughout the world have reported varying findings regarding people's understanding, attitudes, and general awareness of dental implants [26-30]. The acceptance and popularity of dental implants as a treatment option may be connected to the vast differences in dental implant knowledge between various nations. In Austria, 64% of patients were aware of implant treatments [31], followed by 27.7% in Turkey [32], 23.24% in India [33], and 66.4% in Saudi Arabia [13], according to studies looking at patient comprehension of dental implants globally. The American public has a high level of awareness of and favorable perceptions of dental implant therapy [34]. The way the public views and feels about implant therapy depends on the information source. The mass media is more likely to report on implant failures and malpractice, which could lower public support for the procedure. On the other hand, information from experts in dentistry and patients who have had implants may change how the public views and supports the procedure [35].

The clinical implication of the study tells that knowledge and awareness are tested in this study for the population which can help to generate a list of the points where the knowledge and awareness about dental implantology can be improved amongst the selected population. By telling about the benefits of the treatment by not having any effects on the adjacent tooth when we go for fixed partial dentures, we could increase the awareness amongst the individuals and so also insurance can be advocated amongst the middle-class people so that they can have the benefits of the treatment.

The limitations of the study are a very small sample population and consideration of only one geographical area. The findings of this research need to be confirmed by larger population investigations. Less awareness and information regarding implants is learned through literature or from the patient's dentist. As they play a significant role in further educating their patients about treatment alternatives, it is thought that brief courses should be offered to boost their understanding regarding dental implants.

## Conclusions

Although the information regarding dental implants obtained from the study population is general, a number of conclusions can be drawn from our study. For starters, over the study population's lifespan, there has been a shift in the source of information from the internet, social media, and family and friends to dentists. It was also observed that there is inadequate knowledge regarding the longevity of dental implants, and participants who were working in the government sector had dental implants and were aware that dental implants as a treatment option were provided by their dentist when compared to those who were working in the private sector, and around half are not aware that dental implant treatment can be covered by insurance. Moreover, the results of our study also emphasized the need for providing more general and correct information to patients about dental implants and the substantial need for providing community-based awareness programs regarding the knowledge and awareness of dental implants by undergraduate and postgraduate dental students.
